# Supplementary material for: Nuclear Gene Transformation in the Dinoflagellate Oxyrrhis marina
Source: Microorganisms. 2020 Jan 16;8(1):126. doi: 10.3390/microorganisms8010126 (PMC7022241; doi:10.3390/microorganisms8010126)
Supplement: Supplementary file 1 [file microorganisms-08-00126-s001.zip › Micro_Sprecher-et-al/Micro_Sprecher et al._Supplemental.docx]

Supporting Online Material

Nuclear gene transformation in the dinoflagellate *Oxyrrhis marina*

Brittany N. Sprecher, Huan Zhang*, Senjie Lin*

* Address for correspondence; emails [senjie.lin@uconn.edu](mailto:senjie.lin@uconn.edu); [huan.zhang@uconn.edu](mailto:huan.zhang@uconn.edu)

**Supplementary Video Legend**

**Supplementary Video 1a:** *Oxyrrhis marina* cells transformed with *gfp* showing green florescence under blue light. Video was taking using Olympus BX51 microscopic system with a DP74 Olympus camera under 400X magnification.

**Supplementary Video 1b:** *Oxyrrhis marina* cell transformed with *gfp* showing green florescence under blue light and regular emitted light. Video was taking using Olympus BX51 microscopic system with a DP74 Olympus camera under 400X magnification.

**Supplementary Video 2:** *Oxyrrhis marina* DinoIII pulse control wild-type cells under blue light and regular emitted light showing no fluorescence. Video was taking using Olympus BX51 microscopic system with a DP74 Olympus camera under 400X magnification.

**Supplementary Figures**

**Figure S1:** Structure of the DinoIII-*gfp*. The bacterial pMD-19TM T-Vector portion is shown in brown, while other colors depict dinoflagellate elements, including the DinoSL Complex region (purple; containing *K. brevis* SL RNA, SRP RNA, several tRNAs, and U6 region) and the Promotor (light green) and Termination (light blue) Regions, which are the upstream and downstream regions of *F. kawagutii* light harvesting complex, respectively. The GFP region is shown in green. *Eco*RI site depicts where DinoIII-*gfp* is digested to form linear plasmid DNA.

**Figure S2:** Structure of the DinoIII-*arrO*. The bacterial pMD-19TM T-Vector portion is shown in brown, while other colors depict dinoflagellate elements, including the DinoSL Complex region (purple; containing *K. brevis* SL RNA, SRP RNA, several tRNAs, and U6 region) and the Promotor (light green) and Termination (light blue) Regions, which are the upstream and downstream regions of *F. kawagutii* light harvesting complex, respectively. The *arrO* region is shown in pink.

**Figure S3:** Structure of the DinoIII-*arrO*-N. The bacterial pMD-19TM T-Vector portion is shown in brown, while other colors depict dinoflagellate elements, including the DinoSL Complex region (purple; containing *K. brevis* SL RNA, SRP RNA, several tRNAs, and U6 region) and the Promotor (light green) and Termination (light blue) Regions, which are the upstream and downstream regions of *F. kawagutii* light harvesting complex, respectively. The *arrO* region is shown in pink and the interspacer region, a darker pink, is depicted by a line.

**Figure S4:** *Oxyrrhis marina* cell counts under different rifampin concentrations (in μg/mL).

**Figure S5:** *Oxyrrhis marina* relative cell survival under different rifampin concentrations (in μg/mL) for wild-type (WT) versus transformed, +*arrO*-N, conditions. Cells were fed additional *D. tertiolecta* on day 3 and note that on the final day only *arrO*-N in 225 μg/mL still had *D. tertiolecta* present.

**Figure S6:** Wild-type *O. marina* cell counts under 225 μg/mL rifampin after begin fed transformed *D. tertiolecta* (+*arrO*/*arrO*-N). The conditions refer to the different pulse codes used to transform *D. tertiolecta.*

**Supplementary Tables**

**Table S1:** The RNA complex sequence from the dinoflagellate *Karenia brevis* (GenBank accession # FJ434727) containing SL RNA, SRP RNA, several tRNAs, and U6. (Sequence highlighted in yellow).

| Name | Sequence |
| --- | --- |
| RNA Elements | CCCGTAGCCATTTTGGCTCAAGGTACAAGTCGGGCTGATGCGGTCACGCAGGCCTCTTTTGTATCAGATAAGACAGCAGACGTACATGATATAAATATTTATTATCGCGATGCTCCATCTGCAAAATTCCACTTGGCAGAGAAGATTTTCAATGCATGAAAACCTCGCAGAACCAAGCACCAATGTCAGATCATGTATGAGTCCTAGGCGCCAGTAAGTCTTTCAATTGGGTGATGGCCACATTACCTGTTCTCTTCCTGAGGGATGCAGAATGTGGTGTGAGTGGTTGCCGAATGCTGTGCTGAACATGTAGATGGGCTGCCCCAGCCAAGTGGATAACCTACAAGGACTGCAGGATGTAGATCCAGCAGTCCTGGTAGATAATGATTCAGACACCGGCTCAGGCTGGCAACAGAGCAGGCAAACCTGAACACTATCGCGCAGTGGTGGGAATACAGCCCAGAATGGGAAGCAACCTGGCCTCTTCAGCCAGCCATGACCACAAGCAACTCTGCAGTTTTGCAACACGAAATCTTTCAGGGTCTGCTCCCTGTGCTTACACATATTCAAAGATATTCTTCTGCGCATTTCAATGCAAATTCTTGGCGATGATTCGCTTCCGCAGGTATAGTGTTACTAGTATATAAGTCTAATCATATACTTCAATTTATTAGTAATATTGTGAATTTCTAGGCAAGAAGGATGCCTGGACTCTGAATTATTATGGCATTGAGTAGAATCTGGATCTTGATGATTATGCATTAATATCTTGAAATGCATTTGGATTCCCTTCGGGGATCATCCGTTAAAATTGGAACGATACAGAGAAGATTAGCATGGCCCCTGCGCAAGGATGACACGCACAAATCGAGAAGTGTAAACAATTTTTTTGAAATTAATTGCCACTTTATTTTGAATACCTGAATATGCAGGTGAAGTAGTATAAGGTATTCATTATCATTGCGATTTTCTAG |

**Table S2**: The “promoter” region sequence containing the upstream region of the highly expressed light harvesting complex (LHC) gene from the dinoflagellate *Fugacium kawagutii* (formerly *Symbiodinium kawagutii*)*.* (Sequence highlighted in blue).

| Name | Sequence |
| --- | --- |
| Promoter Region | TCCCGTGGCTGTCATATCTAGTAACCCTCACCTGGCAGGTGGGGAAAAGGCGAAGACAAATAGAATCAAATAGAATGTATGTGCTGACGTAGGGCTACATTACCTGAGGCTTGAGGAACCTGTGGGTATGAATGCTACTGTTGGCAGCATCCAGTAGGTTCAGAAACACACGTCCCCACGTAATTTTTTGCATGTCTAAATGCACTGTGATGATTGATGATGATTGATTCCTTGAAGGCCATGGTGGGTTACTCTGTGGCCTCTTGCCACTGACTTCCAGGAAAACGCGGATTTTCTGACCATCACAAGGGACCACTACGAGAAGTATGCAGCGATCTCAGCAGAGTTCATTTACTGACCATTCTCAGGAAGATCGCTCGGGATCACTTGGGTAATATTGCTTTCTTCGCAGATGTTTTTTCCCTATTAAGCTTTTTGAGTCCCCTCATCGAGTGCCGCATAAGTTTTTGCGTTGACATTGGCGGCAAAAAGTAAGACTAAACGATAGTTGCTTCAAGCACGCTTCTCAATCAACATTTTTTCAAATCAAATGGGCACAGGCAGGTACCATCCAGTCCCGCAGAAGTGCTTATATATATATATATTATACACTCACGTGAGTGTGTTCAATGCAGGTCTGTTCCTCAAGTGCTTAGACCAAATTTCGGAGCC |

**Table S3:** The “termination” region containing the downstream sequence of the highly expressed light harvesting complex (LHC) gene from the dinoflagellate *Fugacium kawagutii* (formerly *Symbiodinium kawagutii*). (Sequence highlighted in pink).

| Name | Sequence |
| --- | --- |
| Termination Region | CGGCCAGGAGTCACAGAAAACAAGATCACTTGGAGATGTTTCAATCCCGACTTGTGTCGTGCCAGAGTGCTACTTGAAAACTTGAAAATTGCGGACTGTCATGGATTCGCCCTTGTCTTGTGATCCTTTTTTTGGGGGAGCCAGGTGAGAACAATGTTGTCGATGTGCTTATTTGGCTTCGCAGTCAAAACATGGGATACTTGAGACATGAAAGAAAAATGCCGCAACGATAGCTCCATCCAATTCCATTCAGCTCCGACTACAGATGATAGCGCTTGACACCAATGACATGCTTGTACAGCTGCCATTTGGAAGGCAGGGAAGCTCCATAAGCTCGGGTCCCCAGGACTTTGGTCGGTCTCACATCAGATTCGGCTAGCCAGCCCATAGCAGCCGCGGGAGATTTCGGTTGTTTGCTACAATGATTGGGGCGCCTTTCTGCGAACTTTGTGACATGTTTCCTCAAAATGTCAAGCAATTTTGATCTTAAAAGTTTTGATAATGCTTGCTTCCACAAGCGACCTACAGTAGGAAATGTCTCCACAATCTCCACAGATTCAGGACTCATCACTATGTGTGCCGTGCAGGGGTAGGGCGCAGACATGACAACATACAACACACATGAACTAAAGAATCCAAGTCGCGGACAAAAAAATCTGATCTTACACTTACACAGAATGCAGGTTATTAGCGACGCTTCCATTGCCACCGGAGTGGCAATCGTTGAGGCGCTTCATCGAACAGAGGGTGAACTTCTTGAGGCTGGGAGGACCGCGCAGATGCGGCTGATAAAAGTTTCACAAGCACACGGA |

**Table S4:** Dinoflagellate expression vector backbone, containing the pMD^TM^19 T-Vector (shown in lowercase; Takara, Japan), the RNA Elements (highlighted in yellow), the Promoter Region (highlighted in blue), and the Termination Region (highlighted in pink). The restriction enzyme sites for *Xba*I (**tctaga**) and *Bgl*II (**agatct**) shown in bold allow for the easy insertion of any gene of interest in the correct orientation.

| Name | Sequence |
| --- | --- |
| DinoIII | gacgaaagggcctcgtgatacgcctatttttataggttaatgtcatgataataatggtttcttagacgtcaggtggcacttttcggggaaatgtgcgcggaacccctatttgtttatttttctaaatacattcaaatatgtatccgctcatgagacaataaccctgataaatgcttcaataatattgaaaaaggaagagtatgagtattcaacatttccgtgtcgcccttattcccttttttgcggcattttgccttcctgtttttgctcacccagaaacgctggtgaaagtaaaagatgctgaagatcagttgggtgcacgagtgggttacatcgaactggatctcaacagcggtaagatccttgagagttttcgccccgaagaacgttttccaatgatgagcacttttaaagttctgctatgtggcgcggtattatcccgtattgacgccgggcaagagcaactcggtcgccgcatacactattctcagaatgacttggttgagtactcaccagtcacagaaaagcatcttacggatggcatgacagtaagagaattatgcagtgctgccataaccatgagtgataacactgcggccaacttacttctgacaacgatcggaggaccgaaggagctaaccgcttttttgcacaacatgggggatcatgtaactcgccttgatcgttgggaaccggagctgaatgaagccataccaaacgacgagcgtgacaccacgatgcctgtagcaatggcaacaacgttgcgcaaactattaactggcgaactacttactctagcttcccggcaacaattaatagactggatggaggcggataaagttgcaggaccacttctgcgctcggcccttccggctggctggtttattgctgataaatctggagccggtgagcgtgggtctcgcggtatcattgcagcactggggccagatggtaagccctcccgtatcgtagttatctacacgacggggagtcaggcaactatggatgaacgaaatagacagatcgctgagataggtgcctcactgattaagcattggtaactgtcagaccaagtttactcatatatactttagattgatttaaaacttcatttttaatttaaaaggatctaggtgaagatcctttttgataatctcatgaccaaaatcccttaacgtgagttttcgttccactgagcgtcagaccccgtagaaaagatcaaaggatcttcttgagatcctttttttctgcgcgtaatctgctgcttgcaaacaaaaaaaccaccgctaccagcggtggtttgtttgccggatcaagagctaccaactctttttccgaaggtaactggcttcagcagagcgcagataccaaatactgttcttctagtgtagccgtagttaggccaccacttcaagaactctgtagcaccgcctacatacctcgctctgctaatcctgttaccagtggctgctgccagtggcgataagtcgtgtcttaccgggttggactcaagacgatagttaccggataaggcgcagcggtcgggctgaacggggggttcgtgcacacagcccagcttggagcgaacgacctacaccgaactgagatacctacagcgtgagctatgagaaagcgccacgcttcccgaagggagaaaggcggacaggtatccggtaagcggcagggtcggaacaggagagcgcacgagggagcttccagggggaaacgcctggtatctttatagtcctgtcgggtttcgccacctctgacttgagcgtcgatttttgtgatgctcgtcaggggggcggagcctatggaaaaacgccagcaacgcggcctttttacggttcctggccttttgctggccttttgctcacatgttctttcctgcgttatcccctgattctgtggataaccgtattaccgcctttgagtgagctgataccgctcgccgcagccgaacgaccgagcgcagcgagtcagtgagcgaggaagcggaagagcgcccaatacgcaaaccgcctctccccgcgcgttggccgattcattaatgcagctggcacgacaggtttcccgactggaaagcgggcagtgagcgcaacgcaattaatgtgagttagctcactcattaggcaccccaggctttacactttatgcttccggctcgtatgttgtgtggaattgtgagcggataacaatttcacacaggaaacagctatgaccatgattacgccaagcttgcatgcctgcaggtcgacgattCCCGTAGCCATTTTGGCTCAAGGTACAAGTCGGGCTGATGCGGTCACGCAGGCCTCTTTTGTATCAGATAAGACAGCAGACGTACATGATATAAATATTTATTATCGCGATGCTCCATCTGCAAAATTCCACTTGGCAGAGAAGATTTTCAATGCATGAAAACCTCGCAGAACCAAGCACCAATGTCAGATCATGTATGAGTCCTAGGCGCCAGTAAGTCTTTCAATTGGGTGATGGCCACATTACCTGTTCTCTTCCTGAGGGATGCAGAATGTGGTGTGAGTGGTTGCCGAATGCTGTGCTGAACATGTAGATGGGCTGCCCCAGCCAAGTGGATAACCTACAAGGACTGCAGGATGTAGATCCAGCAGTCCTGGTAGATAATGATTCAGACACCGGCTCAGGCTGGCAACAGAGCAGGCAAACCTGAACACTATCGCGCAGTGGTGGGAATACAGCCCAGAATGGGAAGCAACCTGGCCTCTTCAGCCAGCCATGACCACAAGCAACTCTGCAGTTTTGCAACACGAAATCTTTCAGGGTCTGCTCCCTGTGCTTACACATATTCAAAGATATTCTTCTGCGCATTTCAATGCAAATTCTTGGCGATGATTCGCTTCCGCAGGTATAGTGTTACTAGTATATAAGTCTAATCATATACTTCAATTTATTAGTAATATTGTGAATTTCTAGGCAAGAAGGATGCCTGGACTCTGAATTATTATGGCATTGAGTAGAATCTGGATCTTGATGATTATGCATTAATATCTTGAAATGCATTTGGATTCCCTTCGGGGATCATCCGTTAAAATTGGAACGATACAGAGAAGATTAGCATGGCCCCTGCGCAAGGATGACACGCACAAATCGAGAAGTGTAAACAATTTTTTTGAAATTAATTGCCACTTTATTTTGAATACCTGAATATGCAGGTGAAGTAGTATAAGGTATTCATTATCATTGCGATTTTCTAGTAAGTCCCGTGGCTGTCATATCTAGTAACCCTCACCTGGCAGGTGGGGAAAAGGCGAAGACAAATAGAATCAAATAGAATGTATGTGCTGACGTAGGGCTACATTACCTGAGGCTTGAGGAACCTGTGGGTATGAATGCTACTGTTGGCAGCATCCAGTAGGTTCAGAAACACACGTCCCCACGTAATTTTTTGCATGTCTAAATGCACTGTGATGATTGATGATGATTGATTCCTTGAAGGCCATGGTGGGTTACTCTGTGGCCTCTTGCCACTGACTTCCAGGAAAACGCGGATTTTCTGACCATCACAAGGGACCACTACGAGAAGTATGCAGCGATCTCAGCAGAGTTCATTTACTGACCATTCTCAGGAAGATCGCTCGGGATCACTTGGGTAATATTGCTTTCTTCGCAGATGTTTTTTCCCTATTAAGCTTTTTGAGTCCCCTCATCGAGTGCCGCATAAGTTTTTGCGTTGACATTGGCGGCAAAAAGTAAGACTAAACGATAGTTGCTTCAAGCACGCTTCTCAATCAACATTTTTTCAAATCAAATGGGCACAGGCAGGTACCATCCAGTCCCGCAGAAGTGCTTATATATATATATATTATACACTCACGTGAGTGTGTTCAATGCAGGTCTGTTCCTCAAGTGCTTAGACCAAATTTCGGAGCC**tctagaagatct**CGGCCAGGAGTCACAGAAAACAAGATCACTTGGAGATGTTTCAATCCCGACTTGTGTCGTGCCAGAGTGCTACTTGAAAACTTGAAAATTGCGGACTGTCATGGATTCGCCCTTGTCTTGTGATCCTTTTTTTGGGGGAGCCAGGTGAGAACAATGTTGTCGATGTGCTTATTTGGCTTCGCAGTCAAAACATGGGATACTTGAGACATGAAAGAAAAATGCCGCAACGATAGCTCCATCCAATTCCATTCAGCTCCGACTACAGATGATAGCGCTTGACACCAATGACATGCTTGTACAGCTGCCATTTGGAAGGCAGGGAAGCTCCATAAGCTCGGGTCCCCAGGACTTTGGTCGGTCTCACATCAGATTCGGCTAGCCAGCCCATAGCAGCCGCGGGAGATTTCGGTTGTTTGCTACAATGATTGGGGCGCCTTTCTGCGAACTTTGTGACATGTTTCCTCAAAATGTCAAGCAATTTTGATCTTAAAAGTTTTGATAATGCTTGCTTCCACAAGCGACCTACAGTAGGAAATGTCTCCACAATCTCCACAGATTCAGGACTCATCACTATGTGTGCCGTGCAGGGGTAGGGCGCAGACATGACAACATACAACACACATGAACTAAAGAATCCAAGTCGCGGACAAAAAAATCTGATCTTACACTTACACAGAATGCAGGTTATTAGCGACGCTTCCATTGCCACCGGAGTGGCAATCGTTGAGGCGCTTCATCGAACAGAGGGTGAACTTCTTGAGGCTGGGAGGACCGCGCAGATGCGGCTGATAAAAGTTTCACAAGCACACGGAgaattcactggccgtcgttttacaacgtcgtgactgggaaaaccctggcgttacccaacttaatcgccttgcagcacatccccctttcgccagctggcgtaatagcgaagaggcccgcaccgatcgcccttcccaacagttgcgcagcctgaatggcgaatggcgcctgatgcggtattttctccttacgcatctgtgcggtatttcacaccgcatatggtgcactctcagtacaatctgctctgatgccgcatagttaagccagccccgacacccgccaacacccgctgacgcgccctgacgggcttgtctgctcccggcatccgcttacagacaagctgtgaccgtctccgggagctgcatgtgtcagaggttttcaccgtcatcaccgaaacgcgcga |

**Table S5**: DinoIII-*gfp* sequence, containing the pMD^TM^19 T-Vector (shown in lowercase; Takara, Japan), the RNA Elements (highlighted in yellow), the Promoter Region (highlighted in blue), GFP gene (highlighted in green), and the Termination Region (highlighted in pink). Sequence homology of the green fluorescent protein from the crystal jelly, *Aequorea Victoria.* Sequence was obtained from the pGlo^TM^ Plasmid (Bio-Rad, USA). GenBank: U62637.1.

| Name | Sequence |
| --- | --- |
| DinoIII-*gfp* | gacgaaagggcctcgtgatacgcctatttttataggttaatgtcatgataataatggtttcttagacgtcaggtggcacttttcggggaaatgtgcgcggaacccctatttgtttatttttctaaatacattcaaatatgtatccgctcatgagacaataaccctgataaatgcttcaataatattgaaaaaggaagagtatgagtattcaacatttccgtgtcgcccttattcccttttttgcggcattttgccttcctgtttttgctcacccagaaacgctggtgaaagtaaaagatgctgaagatcagttgggtgcacgagtgggttacatcgaactggatctcaacagcggtaagatccttgagagttttcgccccgaagaacgttttccaatgatgagcacttttaaagttctgctatgtggcgcggtattatcccgtattgacgccgggcaagagcaactcggtcgccgcatacactattctcagaatgacttggttgagtactcaccagtcacagaaaagcatcttacggatggcatgacagtaagagaattatgcagtgctgccataaccatgagtgataacactgcggccaacttacttctgacaacgatcggaggaccgaaggagctaaccgcttttttgcacaacatgggggatcatgtaactcgccttgatcgttgggaaccggagctgaatgaagccataccaaacgacgagcgtgacaccacgatgcctgtagcaatggcaacaacgttgcgcaaactattaactggcgaactacttactctagcttcccggcaacaattaatagactggatggaggcggataaagttgcaggaccacttctgcgctcggcccttccggctggctggtttattgctgataaatctggagccggtgagcgtgggtctcgcggtatcattgcagcactggggccagatggtaagccctcccgtatcgtagttatctacacgacggggagtcaggcaactatggatgaacgaaatagacagatcgctgagataggtgcctcactgattaagcattggtaactgtcagaccaagtttactcatatatactttagattgatttaaaacttcatttttaatttaaaaggatctaggtgaagatcctttttgataatctcatgaccaaaatcccttaacgtgagttttcgttccactgagcgtcagaccccgtagaaaagatcaaaggatcttcttgagatcctttttttctgcgcgtaatctgctgcttgcaaacaaaaaaaccaccgctaccagcggtggtttgtttgccggatcaagagctaccaactctttttccgaaggtaactggcttcagcagagcgcagataccaaatactgttcttctagtgtagccgtagttaggccaccacttcaagaactctgtagcaccgcctacatacctcgctctgctaatcctgttaccagtggctgctgccagtggcgataagtcgtgtcttaccgggttggactcaagacgatagttaccggataaggcgcagcggtcgggctgaacggggggttcgtgcacacagcccagcttggagcgaacgacctacaccgaactgagatacctacagcgtgagctatgagaaagcgccacgcttcccgaagggagaaaggcggacaggtatccggtaagcggcagggtcggaacaggagagcgcacgagggagcttccagggggaaacgcctggtatctttatagtcctgtcgggtttcgccacctctgacttgagcgtcgatttttgtgatgctcgtcaggggggcggagcctatggaaaaacgccagcaacgcggcctttttacggttcctggccttttgctggccttttgctcacatgttctttcctgcgttatcccctgattctgtggataaccgtattaccgcctttgagtgagctgataccgctcgccgcagccgaacgaccgagcgcagcgagtcagtgagcgaggaagcggaagagcgcccaatacgcaaaccgcctctccccgcgcgttggccgattcattaatgcagctggcacgacaggtttcccgactggaaagcgggcagtgagcgcaacgcaattaatgtgagttagctcactcattaggcaccccaggctttacactttatgcttccggctcgtatgttgtgtggaattgtgagcggataacaatttcacacaggaaacagctatgaccatgattacgccaagcttgcatgcctgcaggtcgacgattCCCGTAGCCATTTTGGCTCAAGGTACAAGTCGGGCTGATGCGGTCACGCAGGCCTCTTTTGTATCAGATAAGACAGCAGACGTACATGATATAAATATTTATTATCGCGATGCTCCATCTGCAAAATTCCACTTGGCAGAGAAGATTTTCAATGCATGAAAACCTCGCAGAACCAAGCACCAATGTCAGATCATGTATGAGTCCTAGGCGCCAGTAAGTCTTTCAATTGGGTGATGGCCACATTACCTGTTCTCTTCCTGAGGGATGCAGAATGTGGTGTGAGTGGTTGCCGAATGCTGTGCTGAACATGTAGATGGGCTGCCCCAGCCAAGTGGATAACCTACAAGGACTGCAGGATGTAGATCCAGCAGTCCTGGTAGATAATGATTCAGACACCGGCTCAGGCTGGCAACAGAGCAGGCAAACCTGAACACTATCGCGCAGTGGTGGGAATACAGCCCAGAATGGGAAGCAACCTGGCCTCTTCAGCCAGCCATGACCACAAGCAACTCTGCAGTTTTGCAACACGAAATCTTTCAGGGTCTGCTCCCTGTGCTTACACATATTCAAAGATATTCTTCTGCGCATTTCAATGCAAATTCTTGGCGATGATTCGCTTCCGCAGGTATAGTGTTACTAGTATATAAGTCTAATCATATACTTCAATTTATTAGTAATATTGTGAATTTCTAGGCAAGAAGGATGCCTGGACTCTGAATTATTATGGCATTGAGTAGAATCTGGATCTTGATGATTATGCATTAATATCTTGAAATGCATTTGGATTCCCTTCGGGGATCATCCGTTAAAATTGGAACGATACAGAGAAGATTAGCATGGCCCCTGCGCAAGGATGACACGCACAAATCGAGAAGTGTAAACAATTTTTTTGAAATTAATTGCCACTTTATTTTGAATACCTGAATATGCAGGTGAAGTAGTATAAGGTATTCATTATCATTGCGATTTTCTAGTAAGTCCCGTGGCTGTCATATCTAGTAACCCTCACCTGGCAGGTGGGGAAAAGGCGAAGACAAATAGAATCAAATAGAATGTATGTGCTGACGTAGGGCTACATTACCTGAGGCTTGAGGAACCTGTGGGTATGAATGCTACTGTTGGCAGCATCCAGTAGGTTCAGAAACACACGTCCCCACGTAATTTTTTGCATGTCTAAATGCACTGTGATGATTGATGATGATTGATTCCTTGAAGGCCATGGTGGGTTACTCTGTGGCCTCTTGCCACTGACTTCCAGGAAAACGCGGATTTTCTGACCATCACAAGGGACCACTACGAGAAGTATGCAGCGATCTCAGCAGAGTTCATTTACTGACCATTCTCAGGAAGATCGCTCGGGATCACTTGGGTAATATTGCTTTCTTCGCAGATGTTTTTTCCCTATTAAGCTTTTTGAGTCCCCTCATCGAGTGCCGCATAAGTTTTTGCGTTGACATTGGCGGCAAAAAGTAAGACTAAACGATAGTTGCTTCAAGCACGCTTCTCAATCAACATTTTTTCAAATCAAATGGGCACAGGCAGGTACCATCCAGTCCCGCAGAAGTGCTTATATATATATATATTATACACTCACGTGAGTGTGTTCAATGCAGGTCTGTTCCTCAAGTGCTTAGACCAAATTTCGGAGCCtctagtATGGCTAGCAAAGGAGAAGAACTTTTCACTGGAGTTGTCCCAATTCTTGTTGAATTAGATGGTGATGTTAATGGGCACAAATTTTCTGTCAGTGGAGAGGGTGAAGGTGATGCTACATACGGAAAGCTTACCCTTAAATTTATTTGCACTACTGGAAAACTACCTGTTCCATGGCCAACACTTGTCACTACTTTCTCTTATGGTGTTCAATGCTTTTCCCGTTATCCGGATCATATGAAACGGCATGACTTTTTCAAGAGTGCCATGCCCGAAGGTTATGTACAGGAACGCACTATATCTTTCAAAGATGACGGGAACTACAAGACGCGTGCTGAAGTCAAGTTTGAAGGTGATACCCTTGTTAATCGTATCGAGTTAAAAGGTATTGATTTTAAAGAAGATGGAAACATTCTCGGACACAAACTCGAGTACAACTATAACTCACACAATGTATACATCACGGCAGACAAACAAAAGAATGGAATCAAAGCTAACTTCAAAATTCGCCACAACATTGAAGATGGATCCGTTCAACTAGCAGACCATTATCAACAAAATACTCCAATTGGCGATGGCCCTGTCCTTTTACCAGACAACCATTACCTGTCGACACAATCTGCCCTTTCGAAAGATCCCAACGAAAAGCGTGACCACATGGTCCTTCTTGAGTTTGTAACTGCTGCTGGGATTACACATGGCATGGATGAGCTCTACAAATGAtgatctCGGCCAGGAGTCACAGAAAACAAGATCACTTGGAGATGTTTCAATCCCGACTTGTGTCGTGCCAGAGTGCTACTTGAAAACTTGAAAATTGCGGACTGTCATGGATTCGCCCTTGTCTTGTGATCCTTTTTTTGGGGGAGCCAGGTGAGAACAATGTTGTCGATGTGCTTATTTGGCTTCGCAGTCAAAACATGGGATACTTGAGACATGAAAGAAAAATGCCGCAACGATAGCTCCATCCAATTCCATTCAGCTCCGACTACAGATGATAGCGCTTGACACCAATGACATGCTTGTACAGCTGCCATTTGGAAGGCAGGGAAGCTCCATAAGCTCGGGTCCCCAGGACTTTGGTCGGTCTCACATCAGATTCGGCTAGCCAGCCCATAGCAGCCGCGGGAGATTTCGGTTGTTTGCTACAATGATTGGGGCGCCTTTCTGCGAACTTTGTGACATGTTTCCTCAAAATGTCAAGCAATTTTGATCTTAAAAGTTTTGATAATGCTTGCTTCCACAAGCGACCTACAGTAGGAAATGTCTCCACAATCTCCACAGATTCAGGACTCATCACTATGTGTGCCGTGCAGGGGTAGGGCGCAGACATGACAACATACAACACACATGAACTAAAGAATCCAAGTCGCGGACAAAAAAATCTGATCTTACACTTACACAGAATGCAGGTTATTAGCGACGCTTCCATTGCCACCGGAGTGGCAATCGTTGAGGCGCTTCATCGAACAGAGGGTGAACTTCTTGAGGCTGGGAGGACCGCGCAGATGCGGCTGATAAAAGTTTCACAAGCACACGGAgaattcactggccgtcgttttacaacgtcgtgactgggaaaaccctggcgttacccaacttaatcgccttgcagcacatccccctttcgccagctggcgtaatagcgaagaggcccgcaccgatcgcccttcccaacagttgcgcagcctgaatggcgaatggcgcctgatgcggtattttctccttacgcatctgtgcggtatttcacaccgcatatggtgcactctcagtacaatctgctctgatgccgcatagttaagccagccccgacacccgccaacacccgctgacgcgccctgacgggcttgtctgctcccggcatccgcttacagacaagctgtgaccgtctccgggagctgcatgtgtcagaggttttcaccgtcatcaccgaaacgcgcga |

**Table S6:** DinoIII-*arrO* sequence, containing the pMD^TM^19 T-Vector (shown in lowercase; Takara, Japan), the RNA Elements (highlighted in yellow), the Promoter Region (highlighted in blue), *arrO* gene (highlighted in teal), and the Termination Region (highlighted in pink). The *arrO* gene is an *Oxyrrhis marina* codon optimized homolog of the Rifampin ADP-ribosylating transferase gene from the bacterium *Citrobacter freundii* (GenBank accession # NC_019991).

| Name | Sequence |
| --- | --- |
| DinoIII-*arrO* | gacgaaagggcctcgtgatacgcctatttttataggttaatgtcatgataataatggtttcttagacgtcaggtggcacttttcggggaaatgtgcgcggaacccctatttgtttatttttctaaatacattcaaatatgtatccgctcatgagacaataaccctgataaatgcttcaataatattgaaaaaggaagagtatgagtattcaacatttccgtgtcgcccttattcccttttttgcggcattttgccttcctgtttttgctcacccagaaacgctggtgaaagtaaaagatgctgaagatcagttgggtgcacgagtgggttacatcgaactggatctcaacagcggtaagatccttgagagttttcgccccgaagaacgttttccaatgatgagcacttttaaagttctgctatgtggcgcggtattatcccgtattgacgccgggcaagagcaactcggtcgccgcatacactattctcagaatgacttggttgagtactcaccagtcacagaaaagcatcttacggatggcatgacagtaagagaattatgcagtgctgccataaccatgagtgataacactgcggccaacttacttctgacaacgatcggaggaccgaaggagctaaccgcttttttgcacaacatgggggatcatgtaactcgccttgatcgttgggaaccggagctgaatgaagccataccaaacgacgagcgtgacaccacgatgcctgtagcaatggcaacaacgttgcgcaaactattaactggcgaactacttactctagcttcccggcaacaattaatagactggatggaggcggataaagttgcaggaccacttctgcgctcggcccttccggctggctggtttattgctgataaatctggagccggtgagcgtgggtctcgcggtatcattgcagcactggggccagatggtaagccctcccgtatcgtagttatctacacgacggggagtcaggcaactatggatgaacgaaatagacagatcgctgagataggtgcctcactgattaagcattggtaactgtcagaccaagtttactcatatatactttagattgatttaaaacttcatttttaatttaaaaggatctaggtgaagatcctttttgataatctcatgaccaaaatcccttaacgtgagttttcgttccactgagcgtcagaccccgtagaaaagatcaaaggatcttcttgagatcctttttttctgcgcgtaatctgctgcttgcaaacaaaaaaaccaccgctaccagcggtggtttgtttgccggatcaagagctaccaactctttttccgaaggtaactggcttcagcagagcgcagataccaaatactgttcttctagtgtagccgtagttaggccaccacttcaagaactctgtagcaccgcctacatacctcgctctgctaatcctgttaccagtggctgctgccagtggcgataagtcgtgtcttaccgggttggactcaagacgatagttaccggataaggcgcagcggtcgggctgaacggggggttcgtgcacacagcccagcttggagcgaacgacctacaccgaactgagatacctacagcgtgagctatgagaaagcgccacgcttcccgaagggagaaaggcggacaggtatccggtaagcggcagggtcggaacaggagagcgcacgagggagcttccagggggaaacgcctggtatctttatagtcctgtcgggtttcgccacctctgacttgagcgtcgatttttgtgatgctcgtcaggggggcggagcctatggaaaaacgccagcaacgcggcctttttacggttcctggccttttgctggccttttgctcacatgttctttcctgcgttatcccctgattctgtggataaccgtattaccgcctttgagtgagctgataccgctcgccgcagccgaacgaccgagcgcagcgagtcagtgagcgaggaagcggaagagcgcccaatacgcaaaccgcctctccccgcgcgttggccgattcattaatgcagctggcacgacaggtttcccgactggaaagcgggcagtgagcgcaacgcaattaatgtgagttagctcactcattaggcaccccaggctttacactttatgcttccggctcgtatgttgtgtggaattgtgagcggataacaatttcacacaggaaacagctatgaccatgattacgccaagcttgcatgcctgcaggtcgacgattCCCGTAGCCATTTTGGCTCAAGGTACAAGTCGGGCTGATGCGGTCACGCAGGCCTCTTTTGTATCAGATAAGACAGCAGACGTACATGATATAAATATTTATTATCGCGATGCTCCATCTGCAAAATTCCACTTGGCAGAGAAGATTTTCAATGCATGAAAACCTCGCAGAACCAAGCACCAATGTCAGATCATGTATGAGTCCTAGGCGCCAGTAAGTCTTTCAATTGGGTGATGGCCACATTACCTGTTCTCTTCCTGAGGGATGCAGAATGTGGTGTGAGTGGTTGCCGAATGCTGTGCTGAACATGTAGATGGGCTGCCCCAGCCAAGTGGATAACCTACAAGGACTGCAGGATGTAGATCCAGCAGTCCTGGTAGATAATGATTCAGACACCGGCTCAGGCTGGCAACAGAGCAGGCAAACCTGAACACTATCGCGCAGTGGTGGGAATACAGCCCAGAATGGGAAGCAACCTGGCCTCTTCAGCCAGCCATGACCACAAGCAACTCTGCAGTTTTGCAACACGAAATCTTTCAGGGTCTGCTCCCTGTGCTTACACATATTCAAAGATATTCTTCTGCGCATTTCAATGCAAATTCTTGGCGATGATTCGCTTCCGCAGGTATAGTGTTACTAGTATATAAGTCTAATCATATACTTCAATTTATTAGTAATATTGTGAATTTCTAGGCAAGAAGGATGCCTGGACTCTGAATTATTATGGCATTGAGTAGAATCTGGATCTTGATGATTATGCATTAATATCTTGAAATGCATTTGGATTCCCTTCGGGGATCATCCGTTAAAATTGGAACGATACAGAGAAGATTAGCATGGCCCCTGCGCAAGGATGACACGCACAAATCGAGAAGTGTAAACAATTTTTTTGAAATTAATTGCCACTTTATTTTGAATACCTGAATATGCAGGTGAAGTAGTATAAGGTATTCATTATCATTGCGATTTTCTAGTAAGTCCCGTGGCTGTCATATCTAGTAACCCTCACCTGGCAGGTGGGGAAAAGGCGAAGACAAATAGAATCAAATAGAATGTATGTGCTGACGTAGGGCTACATTACCTGAGGCTTGAGGAACCTGTGGGTATGAATGCTACTGTTGGCAGCATCCAGTAGGTTCAGAAACACACGTCCCCACGTAATTTTTTGCATGTCTAAATGCACTGTGATGATTGATGATGATTGATTCCTTGAAGGCCATGGTGGGTTACTCTGTGGCCTCTTGCCACTGACTTCCAGGAAAACGCGGATTTTCTGACCATCACAAGGGACCACTACGAGAAGTATGCAGCGATCTCAGCAGAGTTCATTTACTGACCATTCTCAGGAAGATCGCTCGGGATCACTTGGGTAATATTGCTTTCTTCGCAGATGTTTTTTCCCTATTAAGCTTTTTGAGTCCCCTCATCGAGTGCCGCATAAGTTTTTGCGTTGACATTGGCGGCAAAAAGTAAGACTAAACGATAGTTGCTTCAAGCACGCTTCTCAATCAACATTTTTTCAAATCAAATGGGCACAGGCAGGTACCATCCAGTCCCGCAGAAGTGCTTATATATATATATATTATACACTCACGTGAGTGTGTTCAATGCAGGTCTGTTCCTCAAGTGCTTAGACCAAATTTCGGAGCCtctagtATGGTGAAGGATTGGATCCCGATCTCTCACGATAACTACAAGCAGGTGCAGGGACCGTTCTACCACGGAACCAAGGCGAACTTGGCGATCGGAGATTTGCTGACCACCGGCTTCATCTCCCACTTCGAGGACGGACGTATCCTGAAGCACATCTACTTCTCCGCGTTGATGGAGCCGGCTGTGTGGGGAGCTGAGCTGGCTATGTCGCTGTCTGGCTTGGAGGGACGTGGCTACATCTACATCGTGGAGCCGACCGGACCGTTCGAGGACGATCCGAACCTGACCAACAAGAAGTTCCCGGGCAACCCGACCCAGTCCTACCGCACCTGCGAGCCGTTGCGCATCGTGGGCGTGGTGGAGGACTGGGAGGGACACCCGGTGGAGTTGATCCGTGGAATGTTGGACTCGTTGGAGGACTTGAAGCGCCGTGGCTTGCACGTCATCGAGGATTAGtgatctCGGCCAGGAGTCACAGAAAACAAGATCACTTGGAGATGTTTCAATCCCGACTTGTGTCGTGCCAGAGTGCTACTTGAAAACTTGAAAATTGCGGACTGTCATGGATTCGCCCTTGTCTTGTGATCCTTTTTTTGGGGGAGCCAGGTGAGAACAATGTTGTCGATGTGCTTATTTGGCTTCGCAGTCAAAACATGGGATACTTGAGACATGAAAGAAAAATGCCGCAACGATAGCTCCATCCAATTCCATTCAGCTCCGACTACAGATGATAGCGCTTGACACCAATGACATGCTTGTACAGCTGCCATTTGGAAGGCAGGGAAGCTCCATAAGCTCGGGTCCCCAGGACTTTGGTCGGTCTCACATCAGATTCGGCTAGCCAGCCCATAGCAGCCGCGGGAGATTTCGGTTGTTTGCTACAATGATTGGGGCGCCTTTCTGCGAACTTTGTGACATGTTTCCTCAAAATGTCAAGCAATTTTGATCTTAAAAGTTTTGATAATGCTTGCTTCCACAAGCGACCTACAGTAGGAAATGTCTCCACAATCTCCACAGATTCAGGACTCATCACTATGTGTGCCGTGCAGGGGTAGGGCGCAGACATGACAACATACAACACACATGAACTAAAGAATCCAAGTCGCGGACAAAAAAATCTGATCTTACACTTACACAGAATGCAGGTTATTAGCGACGCTTCCATTGCCACCGGAGTGGCAATCGTTGAGGCGCTTCATCGAACAGAGGGTGAACTTCTTGAGGCTGGGAGGACCGCGCAGATGCGGCTGATAAAAGTTTCACAAGCACACGGAgaattcactggccgtcgttttacaacgtcgtgactgggaaaaccctggcgttacccaacttaatcgccttgcagcacatccccctttcgccagctggcgtaatagcgaagaggcccgcaccgatcgcccttcccaacagttgcgcagcctgaatggcgaatggcgcctgatgcggtattttctccttacgcatctgtgcggtatttcacaccgcatatggtgcactctcagtacaatctgctctgatgccgcatagttaagccagccccgacacccgccaacacccgctgacgcgccctgacgggcttgtctgctcccggcatccgcttacagacaagctgtgaccgtctccgggagctgcatgtgtcagaggttttcaccgtcatcaccgaaacgcgcga |

**Table S7:** Codon Optimization table for *Oxyrrhis marina* created from published sequences in GenBank. Highlighted in yellow are codons adopted for the vector.

| Amino Acids | Codon | Number | /1000 | Fraction |
| --- | --- | --- | --- | --- |
| Ala | GCG | 211 | 31.19 | 0.33 |
| Ala | GCA | 76 | 11.23 | 0.12 |
| Ala | GCT | 195 | 28.82 | 0.3 |
| Ala | GCC | 161 | 23.8 | 0.25 |
| Cys | TGT | 22 | 3.25 | 0.2 |
| Cys | TGC | 86 | 12.71 | 0.8 |
| Asp | GAT | 153 | 22.62 | 0.48 |
| Asp | GAC | 169 | 24.98 | 0.52 |
| Glu | GAG | 277 | 40.95 | 0.76 |
| Glu | GAA | 87 | 12.86 | 0.24 |
| Phe | TTT | 66 | 9.76 | 0.23 |
| Phe | TTC | 219 | 32.37 | 0.77 |
| Gly | GGG | 158 | 23.36 | 0.28 |
| Gly | GGA | 122 | 18.03 | 0.22 |
| Gly | GGT | 111 | 16.41 | 0.2 |
| Gly | GGC | 172 | 25.42 | 0.31 |
| His | CAT | 38 | 5.62 | 0.35 |
| His | CAC | 71 | 10.5 | 0.65 |
| Ile | ATA | 7 | 1.03 | 0.02 |
| Ile | ATT | 126 | 18.63 | 0.37 |
| Ile | ATC | 206 | 30.45 | 0.61 |
| Lys | AAG | 407 | 60.16 | 0.88 |
| Lys | AAA | 58 | 8.57 | 0.12 |
| Leu | TTG | 218 | 32.22 | 0.35 |
| Leu | TTA | 7 | 1.03 | 0.01 |
| Leu | CTG | 240 | 35.48 | 0.38 |
| Leu | CTA | 13 | 1.92 | 0.02 |
| Leu | CTT | 73 | 10.79 | 0.12 |
| Leu | CTC | 73 | 10.79 | 0.12 |
| Met | ATG | 203 | 30.01 | 1 |
| Asn | AAT | 57 | 8.43 | 0.26 |
| Asn | AAC | 165 | 24.39 | 0.74 |
| Pro | CCG | 109 | 16.11 | 0.41 |
| Pro | CCA | 43 | 6.36 | 0.16 |
| Pro | CCT | 58 | 8.57 | 0.22 |
| Pro | CCC | 59 | 8.72 | 0.22 |
| Gln | CAG | 209 | 30.89 | 0.83 |
| Gln | CAA | 42 | 6.21 | 0.17 |
| Arg | AGG | 52 | 7.69 | 0.12 |
| Arg | AGA | 25 | 3.7 | 0.06 |
| Arg | CGG | 73 | 10.79 | 0.17 |
| Arg | CGA | 63 | 9.31 | 0.15 |
| Arg | CGT | 95 | 14.04 | 0.22 |
| Arg | CGC | 121 | 17.89 | 0.28 |
| Ser | AGT | 33 | 4.88 | 0.09 |
| Ser | AGC | 75 | 11.09 | 0.2 |
| Ser | TCG | 91 | 13.45 | 0.25 |
| Ser | TCA | 20 | 2.96 | 0.05 |
| Ser | TCT | 59 | 8.72 | 0.16 |
| Ser | TCC | 91 | 13.45 | 0.25 |
| Thr | ACG | 102 | 15.08 | 0.28 |
| Thr | ACA | 47 | 6.95 | 0.13 |
| Thr | ACT | 73 | 10.79 | 0.2 |
| Thr | ACC | 136 | 20.1 | 0.38 |
| Val | GTG | 292 | 43.16 | 0.57 |
| Val | GTA | 23 | 3.4 | 0.04 |
| Val | GTT | 98 | 14.49 | 0.19 |
| Val | GTC | 101 | 14.93 | 0.2 |
| Trp | TGG | 107 | 15.82 | 1 |
| Tyr | TAT | 48 | 7.1 | 0.26 |
| Tyr | TAC | 139 | 20.55 | 0.74 |
| End | TGA | 9 | 1.33 | 0.26 |
| End | TAG | 23 | 3.4 | 0.68 |
| End | TAA | 2 | 0.3 | 0.06 |

**Table S8:** DinoIII-*arrO-*N sequence, containing the pMD^TM^19 T-Vector (shown in lowercase; Takara, Japan), the RNA Elements (highlighted in yellow), the Promoter Region (highlighted in blue), *arrO-*N gene (highlighted in teal), and the Termination Region (highlighted in pink). The *arrO* gene is an *Oxyrrhis marina* codon optimized homolog of a Rifampin ADP-ribosylating transferase gene from the bacterium *Citrobacter freundii* combined with an intergenic region between *O. marina* rhodopsin tandem repeats at the 5’-end (shown in lowercase and highlighted in teal).

| Name | Sequence |
| --- | --- |
| DinoIII-*arrO-*N | gacgaaagggcctcgtgatacgcctatttttataggttaatgtcatgataataatggtttcttagacgtcaggtggcacttttcggggaaatgtgcgcggaacccctatttgtttatttttctaaatacattcaaatatgtatccgctcatgagacaataaccctgataaatgcttcaataatattgaaaaaggaagagtatgagtattcaacatttccgtgtcgcccttattcccttttttgcggcattttgccttcctgtttttgctcacccagaaacgctggtgaaagtaaaagatgctgaagatcagttgggtgcacgagtgggttacatcgaactggatctcaacagcggtaagatccttgagagttttcgccccgaagaacgttttccaatgatgagcacttttaaagttctgctatgtggcgcggtattatcccgtattgacgccgggcaagagcaactcggtcgccgcatacactattctcagaatgacttggttgagtactcaccagtcacagaaaagcatcttacggatggcatgacagtaagagaattatgcagtgctgccataaccatgagtgataacactgcggccaacttacttctgacaacgatcggaggaccgaaggagctaaccgcttttttgcacaacatgggggatcatgtaactcgccttgatcgttgggaaccggagctgaatgaagccataccaaacgacgagcgtgacaccacgatgcctgtagcaatggcaacaacgttgcgcaaactattaactggcgaactacttactctagcttcccggcaacaattaatagactggatggaggcggataaagttgcaggaccacttctgcgctcggcccttccggctggctggtttattgctgataaatctggagccggtgagcgtgggtctcgcggtatcattgcagcactggggccagatggtaagccctcccgtatcgtagttatctacacgacggggagtcaggcaactatggatgaacgaaatagacagatcgctgagataggtgcctcactgattaagcattggtaactgtcagaccaagtttactcatatatactttagattgatttaaaacttcatttttaatttaaaaggatctaggtgaagatcctttttgataatctcatgaccaaaatcccttaacgtgagttttcgttccactgagcgtcagaccccgtagaaaagatcaaaggatcttcttgagatcctttttttctgcgcgtaatctgctgcttgcaaacaaaaaaaccaccgctaccagcggtggtttgtttgccggatcaagagctaccaactctttttccgaaggtaactggcttcagcagagcgcagataccaaatactgttcttctagtgtagccgtagttaggccaccacttcaagaactctgtagcaccgcctacatacctcgctctgctaatcctgttaccagtggctgctgccagtggcgataagtcgtgtcttaccgggttggactcaagacgatagttaccggataaggcgcagcggtcgggctgaacggggggttcgtgcacacagcccagcttggagcgaacgacctacaccgaactgagatacctacagcgtgagctatgagaaagcgccacgcttcccgaagggagaaaggcggacaggtatccggtaagcggcagggtcggaacaggagagcgcacgagggagcttccagggggaaacgcctggtatctttatagtcctgtcgggtttcgccacctctgacttgagcgtcgatttttgtgatgctcgtcaggggggcggagcctatggaaaaacgccagcaacgcggcctttttacggttcctggccttttgctggccttttgctcacatgttctttcctgcgttatcccctgattctgtggataaccgtattaccgcctttgagtgagctgataccgctcgccgcagccgaacgaccgagcgcagcgagtcagtgagcgaggaagcggaagagcgcccaatacgcaaaccgcctctccccgcgcgttggccgattcattaatgcagctggcacgacaggtttcccgactggaaagcgggcagtgagcgcaacgcaattaatgtgagttagctcactcattaggcaccccaggctttacactttatgcttccggctcgtatgttgtgtggaattgtgagcggataacaatttcacacaggaaacagctatgaccatgattacgccaagcttgcatgcctgcaggtcgacgattCCCGTAGCCATTTTGGCTCAAGGTACAAGTCGGGCTGATGCGGTCACGCAGGCCTCTTTTGTATCAGATAAGACAGCAGACGTACATGATATAAATATTTATTATCGCGATGCTCCATCTGCAAAATTCCACTTGGCAGAGAAGATTTTCAATGCATGAAAACCTCGCAGAACCAAGCACCAATGTCAGATCATGTATGAGTCCTAGGCGCCAGTAAGTCTTTCAATTGGGTGATGGCCACATTACCTGTTCTCTTCCTGAGGGATGCAGAATGTGGTGTGAGTGGTTGCCGAATGCTGTGCTGAACATGTAGATGGGCTGCCCCAGCCAAGTGGATAACCTACAAGGACTGCAGGATGTAGATCCAGCAGTCCTGGTAGATAATGATTCAGACACCGGCTCAGGCTGGCAACAGAGCAGGCAAACCTGAACACTATCGCGCAGTGGTGGGAATACAGCCCAGAATGGGAAGCAACCTGGCCTCTTCAGCCAGCCATGACCACAAGCAACTCTGCAGTTTTGCAACACGAAATCTTTCAGGGTCTGCTCCCTGTGCTTACACATATTCAAAGATATTCTTCTGCGCATTTCAATGCAAATTCTTGGCGATGATTCGCTTCCGCAGGTATAGTGTTACTAGTATATAAGTCTAATCATATACTTCAATTTATTAGTAATATTGTGAATTTCTAGGCAAGAAGGATGCCTGGACTCTGAATTATTATGGCATTGAGTAGAATCTGGATCTTGATGATTATGCATTAATATCTTGAAATGCATTTGGATTCCCTTCGGGGATCATCCGTTAAAATTGGAACGATACAGAGAAGATTAGCATGGCCCCTGCGCAAGGATGACACGCACAAATCGAGAAGTGTAAACAATTTTTTTGAAATTAATTGCCACTTTATTTTGAATACCTGAATATGCAGGTGAAGTAGTATAAGGTATTCATTATCATTGCGATTTTCTAGTAAGTCCCGTGGCTGTCATATCTAGTAACCCTCACCTGGCAGGTGGGGAAAAGGCGAAGACAAATAGAATCAAATAGAATGTATGTGCTGACGTAGGGCTACATTACCTGAGGCTTGAGGAACCTGTGGGTATGAATGCTACTGTTGGCAGCATCCAGTAGGTTCAGAAACACACGTCCCCACGTAATTTTTTGCATGTCTAAATGCACTGTGATGATTGATGATGATTGATTCCTTGAAGGCCATGGTGGGTTACTCTGTGGCCTCTTGCCACTGACTTCCAGGAAAACGCGGATTTTCTGACCATCACAAGGGACCACTACGAGAAGTATGCAGCGATCTCAGCAGAGTTCATTTACTGACCATTCTCAGGAAGATCGCTCGGGATCACTTGGGTAATATTGCTTTCTTCGCAGATGTTTTTTCCCTATTAAGCTTTTTGAGTCCCCTCATCGAGTGCCGCATAAGTTTTTGCGTTGACATTGGCGGCAAAAAGTAAGACTAAACGATAGTTGCTTCAAGCACGCTTCTCAATCAACATTTTTTCAAATCAAATGGGCACAGGCAGGTACCATCCAGTCCCGCAGAAGTGCTTATATATATATATATTATACACTCACGTGAGTGTGTTCAATGCAGGTCTGTTCCTCAAGTGCTTAGACCAAATTTCGGAGCCtctagtaattttgggagttgggctggaagatggggttggtggggatcgggggagaggtgactggtgtgtggtcgagATGGTGAAGGATTGGATCCCGATCTCTCACGATAACTACAAGCAGGTGCAGGGACCGTTCTACCACGGAACCAAGGCGAACTTGGCGATCGGAGATTTGCTGACCACCGGCTTCATCTCCCACTTCGAGGACGGACGTATCCTGAAGCACATCTACTTCTCCGCGTTGATGGAGCCGGCTGTGTGGGGAGCTGAGCTGGCTATGTCGCTGTCTGGCTTGGAGGGACGTGGCTACATCTACATCGTGGAGCCGACCGGACCGTTCGAGGACGATCCGAACCTGACCAACAAGAAGTTCCCGGGCAACCCGACCCAGTCCTACCGCACCTGCGAGCCGTTGCGCATCGTGGGCGTGGTGGAGGACTGGGAGGGACACCCGGTGGAGTTGATCCGTGGAATGTTGGACTCGTTGGAGGACTTGAAGCGCCGTGGCTTGCACGTCATCGAGGATTAGtgatctCGGCCAGGAGTCACAGAAAACAAGATCACTTGGAGATGTTTCAATCCCGACTTGTGTCGTGCCAGAGTGCTACTTGAAAACTTGAAAATTGCGGACTGTCATGGATTCGCCCTTGTCTTGTGATCCTTTTTTTGGGGGAGCCAGGTGAGAACAATGTTGTCGATGTGCTTATTTGGCTTCGCAGTCAAAACATGGGATACTTGAGACATGAAAGAAAAATGCCGCAACGATAGCTCCATCCAATTCCATTCAGCTCCGACTACAGATGATAGCGCTTGACACCAATGACATGCTTGTACAGCTGCCATTTGGAAGGCAGGGAAGCTCCATAAGCTCGGGTCCCCAGGACTTTGGTCGGTCTCACATCAGATTCGGCTAGCCAGCCCATAGCAGCCGCGGGAGATTTCGGTTGTTTGCTACAATGATTGGGGCGCCTTTCTGCGAACTTTGTGACATGTTTCCTCAAAATGTCAAGCAATTTTGATCTTAAAAGTTTTGATAATGCTTGCTTCCACAAGCGACCTACAGTAGGAAATGTCTCCACAATCTCCACAGATTCAGGACTCATCACTATGTGTGCCGTGCAGGGGTAGGGCGCAGACATGACAACATACAACACACATGAACTAAAGAATCCAAGTCGCGGACAAAAAAATCTGATCTTACACTTACACAGAATGCAGGTTATTAGCGACGCTTCCATTGCCACCGGAGTGGCAATCGTTGAGGCGCTTCATCGAACAGAGGGTGAACTTCTTGAGGCTGGGAGGACCGCGCAGATGCGGCTGATAAAAGTTTCACAAGCACACGGAgaattcactggccgtcgttttacaacgtcgtgactgggaaaaccctggcgttacccaacttaatcgccttgcagcacatccccctttcgccagctggcgtaatagcgaagaggcccgcaccgatcgcccttcccaacagttgcgcagcctgaatggcgaatggcgcctgatgcggtattttctccttacgcatctgtgcggtatttcacaccgcatatggtgcactctcagtacaatctgctctgatgccgcatagttaagccagccccgacacccgccaacacccgctgacgcgccctgacgggcttgtctgctcccggcatccgcttacagacaagctgtgaccgtctccgggagctgcatgtgtcagaggttttcaccgtcatcaccgaaacgcgcga |
